# Alpha-Linolenic Acid Intake and 10-Year Incidence of Coronary Heart Disease and Stroke in 20,000 Middle-Aged Men and Women in The Netherlands

**DOI:** 10.1371/journal.pone.0017967

**Published:** 2011-03-25

**Authors:** Janette de Goede, W. M. Monique Verschuren, Jolanda M. A. Boer, Daan Kromhout, Johanna M. Geleijnse

**Affiliations:** 1 Division of Human Nutrition, Wageningen University, Wageningen, The Netherlands; 2 National Institute for Public Health and the Environment, Bilthoven, The Netherlands; University of Swansea, United Kingdom

## Abstract

**Background:**

Whether intake of alpha-linolenic acid (ALA), the plant-derived n-3 polyunsaturated fatty acid (PUFA), could prevent cardiovascular diseases is not yet clear. We examined the associations of ALA intake with 10-year incidence of coronary heart disease (CHD) and stroke in the Netherlands.

**Methods:**

Data were collected from a general population of 20,069 generally healthy men and women, aged 20 to 65 years. Habitual diet was assessed at baseline (1993–1997) with a validated 178-item food frequency questionnaire. Incidences of CHD and stroke were assessed through linkage with mortality and morbidity registers. Hazard ratios (HR) were calculated with multivariable Cox proportional hazards models, adjusted for age, gender, lifestyle, and dietary factors.

**Results:**

During 8–13 years of follow-up, we observed 280 incident CHD events (19% fatal) and 221 strokes (4% fatal). Intakes of energy-adjusted ALA in quintiles ranged from less than 1.0 g/d in the bottom quintile (Q1) to more than 1.9 g/d in the top quintile (Q5). ALA intake was not associated with incident CHD, with HRs varying between 0.89 and 1.01 (all p>0.05) in Q2–Q5 compared with the bottom quintile of ALA intake. For incident stroke, however, participants in Q2–Q5 had a 35–50% lower risk compared with the reference group. HRs were 0.65 (0.43–0.97), 0.49 (0.31–0.76), 0.53 (0.34–0.83), and 0.65 (0.41–1.04) for Q2–Q5 respectively.

**Conclusion:**

In this general Dutch population, ALA intake was not associated with incident CHD. The data suggested that a low intake of ALA may be a risk factor for incident stroke. These results warrant confirmation in other population-based studies and in trials.

## Introduction

Numerous studies suggest that marine n−3 polyunsaturated fatty acids (PUFA), mainly eicosapentaenoic acid (EPA) and docosahexaenoic acid (DHA), protect against cardiovascular diseases (CVD) [1]–[3]. However, the role of the plant-derived n−3 PUFA alpha-linolenic acid (ALA) in CVD prevention is less clear [4]–[6]. ALA is mainly found in vegetable oils such as soybean, canola, and flaxseed oil, and walnuts [7].

In Western countries such as the Netherlands, the intake of ALA is 5–10 times higher than n−3 PUFA from fish [8]. ALA is an essential fatty acid, which means that humans have to obtain it through their diet. Humans can convert ALA into the very-long-chain fatty acids EPA and DHA, although conversion only occurs to a limited extent [9], [10]. Apart from potential indirect effects of ALA on CVD via conversion into EPA and DHA, it is suggested that ALA could have direct anti-inflammatory [6], [11], anti-arrhythmic [12], anti-thrombotic [12] [13], or neuroprotective effects [14]. However, others concluded that there was insufficient evidence that ALA influences risk factors for CVD [15], [16].

Several prospective cohort studies showed inverse associations of ALA intake with fatal CVD [17], fatal coronary heart disease (CHD) [17]–[19], sudden cardiac death [12], incident CHD [20], incident myocardial infarction (MI) [21], or nonfatal MI [20]. Other cohort studies suggested no protection of ALA intake against fatal CVD [22], fatal CHD [12], [21], [23], sudden death [20], incident CHD [23], or nonfatal MI [12], [18]. The relation of ALA intake with fatal CHD has been summarized in a meta-analysis of 5 prospective cohort studies showing that ALA intakes of around 2 g/d were associated with a 21% lower risk of fatal CHD (relative risk: 0.79; 95%CI, 0.60–1.04), compared with intakes of 0.8 g/d [24].

Little is known about the association of ALA intake with stroke. In a nested case-control study in 192 American middle-aged men, serum ALA was inversely associated with stroke [25], although this was not confirmed in a Japanese nested case-control study of with 197 cases of hemorrhagic and ischemic stroke [26]. However, Japanese have higher mortality from stroke and have higher serum levels of n−3 PUFA compared with white Americans and Europeans, which makes it difficult to compare the results [26].

We examined the 10-year incidence of CHD and stroke in relation to ALA intake in a population-based cohort of over 20,000 adults in the Netherlands.

## Methods

### Ethical Statement

This research was performed in accordance with the ethical principles for medical research involving human subjects outlined in the Declaration of Helsinki. This research was approved by the Medical Ethics Committee of TNO Prevention and Health (Leiden, The Netherlands). All participants gave written informed consent.

### Design and study population

The “Monitoring Project on Risk Factors for Chronic Diseases” (MORGEN Study) is a Dutch population-based cohort of 22,654 men and women, aged 20 to 65 years. MORGEN is part of the European Prospective Investigation into Cancer and Nutrition (EPIC) study [27].

For the current study we excluded participants who did not provide informed consent for vital status follow-up (n = 701). We also excluded 72 participants without dietary information and 97 participants with extreme energy intakes (<500 or >4,500 kcal for women and <800 or >5,000 kcal for men). Furthermore, participants with a history of MI or stroke at baseline were excluded (n = 442). We also excluded participants who reported use of lipid or blood pressure lowering medication (n = 1,093) and 180 participants with diabetes resulting in 20,069 participants (8,988 men and 11,081 women).

### Dietary assessment

The habitual diet was assessed at baseline with the Dutch EPIC Food Frequency Questionnaire (FFQ) a self-administered 178-item FFQ covering the previous year [28], [29]. The FFQ included foods that covered the intake of foods and nutrients relevant to chronic disease etiology for at least 90% of the national mean intake. Participants indicated consumption of main food groups in times per day, per week, per month, per year, or as never, combined with questions on the relative intakes of foods within food groups (seldom/never, sometimes, often, mostly/always). In addition, we calculated raw vegetable consumption as the sum of lettuce, cucumber, tomato, carrots, cabbage, sweet pepper, and chicory consumption in grams per day, because these raw vegetables are consumed together with salad dressings, which is a main source of ALA.

Nutrient intakes were calculated with the “Dutch food composition table” of 1998. For individual fatty acids, we used the table of 2001. All nutrients were adjusted for total energy intake with the residuals method [30]. The Dutch EPIC questionnaire has been validated for several food groups and nutrients. The reproducibility (estimated by 2 repeated measurements) and the relative validity (intake assessed by the FFQ compared to intakes assessed by 12 monthly 24-h recalls) of the FFQ for various food groups and nutrients were assessed among 121 Dutch men and women [28], [29]. The Spearman rank correlations for the reproducibility of the FFQ after 6 months were 0.90 and 0.80 for total energy and 0.83 and 0.77 for total fat in men and women respectively. The relative validity of the FFQ was 0.77 and 0.62 for total energy and 0.74 and 0.63 for total fat in men and women respectively.

### Mortality and morbidity

Vital status was checked through linkage with the national population register. Participants were followed for the occurrence of CVD by linkage with Statistics Netherlands for cause-specific mortality and to the national hospital discharge register for nonfatal events by a validated probabilistic linkage method described in detail elsewhere [31]. Incident CHD included fatal CHD (I20–I25), fatal and nonfatal cardiac arrest (I46), and nonfatal MI (I21–I22) according to the International Classification of Diseases (ICD-10, WHO). Incident stroke included fatal and nonfatal cerebrovascular accidents and transient ischemic attacks (I60–I66, G45). For hospital admissions, corresponding ICD9 codes were used. Both primary and secondary causes of death were used for the classification of fatal events. For nonfatal events we used the primary indication for hospital admission. Participants were followed until death, incident CHD or stroke (first events only), date of loss-to-follow-up (n = 693) or 1 January 2006, whichever came first.

### Other baseline characteristics

Body weight, height, and blood pressure were measured by trained research nurses. Levels of total cholesterol and high-density lipoprotein cholesterol were assessed in plasma (non-fasting) [32]. Questionnaires were used to assess presence of diabetes, history of MI, history of stroke, medication use, parental history of MI (MI of father before the age of 55 year or MI of mother before the age of 65 years), educational level, and cigarette smoking. Alcohol intake (assessed by FFQ) was categorized as no intake, low to moderate intake (men≤2 and women≤1 glasses/d), or high intake (men>2 and women>1 glasses/d). Physical activity was assessed with a validated questionnaire in 76% of our cohort (from 1994 onwards) [33]. For this subset, we calculated whether participants were engaged in activities with a metabolic equivalent score ≥4 (yes/no). Cycling (yes/no) and sports (yes/no) were previously shown to be inversely related to CVD incidence in this study population [34].

### Statistical analysis

Participants' characteristics by quintiles of energy-adjusted ALA intake are presented as means with SD, medians with interquartile ranges, or percentages. Correlations between the energy-adjusted intakes of different types of fatty acids were assessed with the Spearman rank correlation test.

We used Cox proportional hazards models to estimate relative risks for the incidence of CHD, total stroke, and ischemic stroke across quintiles of energy-adjusted ALA intake at baseline. For hemorrhagic stroke we had insufficient cases. Hazard ratios (HR) with 95% confidence intervals (CI) were obtained with the bottom quintile of ALA intake as the reference category. The proportional hazards assumption was tested and not rejected based on Schoenfeld residuals and visual inspection.

In model 1, we adjusted for age and gender. In model 2, we additionally adjusted for total energy intake (kJ/d), body mass index (kg/m^2^), alcohol intake (no, low to moderate, or high), current cigarette smoking, high educational level (completed higher vocational training or university), parental history of MI. In model 3, we added energy-adjusted intakes of vitamin C (mg/d), beta-carotene (µg/d), fiber (g/d), saturated fatty acids (g/d), trans fatty acids (g/d), and polyunsaturated fatty acids other than ALA (g/d).

Possible confounding by physical activity was checked in the subgroup of participants with information on physical activity. We examined whether further adjustment for systolic blood pressure and total cholesterol changed the association of ALA with CHD and stroke to assess whether these factors could be intermediates. Effect modification was evaluated for age and gender. In various foods ALA is highly correlated with saturated fatty acids and trans fatty acids. We therefore separately analyzed ALA intake from salad dressings (mayonnaise+soy bean oil), with a low content of saturated fatty acids and trans fatty acids vs. ALA intake from other sources, mutually adjusted. These analyses were additionally adjusted for the intake of raw vegetables. All statistical analyses were performed with SAS (version 9.1; SAS Institute). Two-sided p-values<0.05 were considered statistically significant.

## Results

### Population characteristics

Participants were on average ± SD 41.5±11.1 years at baseline, and 45% were male. Men had higher ALA intakes than women (1.6±0.6 vs. 1.2±0.5 g), but values were similar after energy adjustment (1.4±0.4 g). During 8–13 years of follow-up (median 10.5 y), 280 CHD events (19% fatal) and 221 strokes (4% fatal) occurred. Total stroke comprised 80 cases of ischemic cerebrovascular accident (36%), 59 transient ischemic attacks (27%), 47 cases of hemorrhagic stroke (21%), and 35 cases of unspecified stroke (16%).

The main sources of ALA intake were mayonnaise (15%), margarine (14%), soy bean oil (8%), and bread (8%). Median energy-adjusted ALA intakes in quintiles ranged from less than 1.0 g/d to more than 1.9 g/d. ALA intake was positively associated with intakes of total PUFA (mainly linoleic acid), cis-monounsaturated fat, trans fatty acids, and saturated fatty acids, but not with EPA+DHA (**Table 1**). The Spearman rank correlations with ALA were 0.54 for linoleic acid, 0.41 for cis-monounsaturated fatty acids, 0.22 for trans fatty acids, and 0.18 for saturated fatty acids.

**Table 1:** Baseline characteristics of 20,069 Dutch men and women, aged 20–65 year, by quintiles of energy-adjusted ALA intake^1^.

	Quintiles of ALA intake, g/d				
	Q1	Q2	Q3	Q4	Q5
N	4,013	4,014	4,014	4,014	4,014
ALA, g/d	0.9±0.2	1.2±0.1	1.3±0.05	1.5±0.1	2.0±0.4
ALA, % of energy	0.4±0.05	0.4±0.03	0.5±0.02	0.6±0.04	0.8±0.1
ALA from dressings, g/d	0.1±0.1	0.3±0.1	0.3±0.2	0.4±0.2	0.7±0.5
ALA from other sources, g/d	0.8±0.2	0.9±0.1	1.0±0.2	1.1±0.2	1.3±0.4
Linoleic acid, g/d	11.1±4.3	12.5±3.2	13.4±3.0	14.6±3.1	17.2±4.2
Linoleic acid, % of energy	4.4±1.4	4.8±1.3	5.2±1.2	5.8±1.2	6.7±1.5
EPA+DHA,^2^ mg/d	114 (61–194)	110 (60–192)	111 (58–189)	112 (61–194)	117 (65–198)
Male gender, %	59	41	38	40	46
Age, y	41.8±11.7	42.0±11.2	41.8±11.0	41.3±10.9	40.6±10.6
Body mass index, kg/m^2^	24.9±3.7	24.9±3.8	24.8±3.8	24.8±3.8	24.9±4.1
Polyunsaturated fatty acids, g/d	13.9±4.40	15.9±3.2	17.0±3.0	18.4±3.1	21.6±4.3
Polyunsaturated fatty acids, % of energy	5.6±1.5	6.1±1.3	6.6±1.2	7.3±1.2	8.4±1.5
Cis-monounsaturated fatty acids, g/d	27.5±5.9	30.0±4.7	31.1±4.6	32.0±4.7	34.0±5.8
Cis-monounsaturated fatty acids, % of energy	10.9±2.0	11.5±1.9	12.0±1.9	12.5±1.9	13.3±2.0
Trans fatty acids, g/d	3.4±1.5	3.7±1.2	3.8±1.1	3.9±1.2	4.1±1.5
Trans fatty acids, % of energy	1.4±0.5	1.4±0.5	1.5±0.5	1.5±0.5	1.6±0.5
Saturated fatty acids, g/d	35.1±7.8	36.7±6.1	37.3±5.8	37.7±5.8	38.3±6.4
Saturated fatty acids, % of energy	13.8±2.7	14.2±2.5	14.4±2.4	14.6±2.4	14.9±2.4
Carbohydrate, % of energy	45.4±6.3	44.5±5.8	43.5±5.4	42.7±5.2	41.3±5.1
Protein, % of energy	15.0±2.4	15.5±2.3	15.3±2.2	15.2±2.1	14.4±2.0
Vitamin C,^2^ mg/d	103 (77–138)	101 (77–132)	99 (77–129)	98 (75–127)	93 (70–124)
Beta carotene, mg/d ^†^	1.4 (1.1–1.7)	1.4 (1.1–1.8)	1.4 (1.2–1.8)	1.5 (1.2–1.9)	1.5 (1.2–2.0)
Fiber, g/d	24.3±6.0	24.7±5.1	24.7±4.8	24.8±4.9	24.5±5.4
Energy intake, MJ/d	10.6±2.9	9.0±2.5	8.8±2.5	9.1±2.6	10.1±2.9
Current smoking, %	34	33	35	38	44
Alcohol consumption, %					
No	12	13	12	12	13
Low to moderate	50	58	58	61	58
High	38	29	30	27	29
Highly educated,^3^ %	24	25	27	25	24
Dutch ethnicity, %	97	97	97	96	95
Physically active,^4^ %					
Engaged in cycling	59	61	60	59	57
Engaged in sports	39	40	38	35	34
Parental history of myocardial infarction, %	8	9	9	10	9
Plasma total cholesterol,^5^ mmol/l	5.3±1.1	5.3±1.0	5.3±1.1	5.3±1.1	5.2±1.0
Plasma HDL-cholesterol,^5^ mmol/l	1.3±0.4	1.4±0.4	1.4±0.4	1.4±0.4	1.3±0.4
Systolic blood pressure, mm Hg	122.0±15.7	120.1±15.9	119.5±15.5	119.0±15.1	119.0±15.3
Diastolic blood pressure, mm Hg	77.0±10.4	76.2±10.4	76.0±10.5	75.7±10.2	75.6±10.3

Footnotes Table 1.

ALA: alpha-linolenic acid; Q1–Q5: quintiles; EPA: eicosapentaenoic acid; DHA: docosahexaenoic acid.

1 Values are means ± SD, unless indicated otherwise.

2 Median with interquartile range.

3 University or higher vocational training.

4 Available for participants enrolled between 1994 and 1997 (n = 15,423).

5 Nonfasting.

### ALA intake and incident CHD and stroke

**Figure 1:**
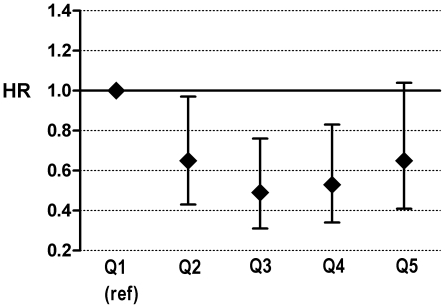
The association of incident total stroke by quintiles of energy-adjusted ALA intake ^1,2^. ^1^ Hazard ratios (95% CI) with the first quintile as the reference category, adjusted for age, gender, body mass index total energy intake, alcohol intake, cigarette smoking, education level, parental history of myocardial infarction, intake of vitamin C, beta-carotene, fiber, saturated fatty acids, trans fatty acids, polyunsaturated fatty acids other than ALA. ^2^ ALA: alpha-linolenic acid; Q1–Q5: quintiles.

After adjustment for potential confounders, ALA intake was not associated with incident CHD. HRs varied between 0.89 and 1.01 (all p>0.05) compared with the bottom quintile of ALA intake (**Table 2**). ALA intake was inversely associated with total stroke (Figure 1) and ischemic stroke incidence. Compared with the lowest quintile of ALA intake (<1.1 g/d), participants in the other quintiles had a 35–50% lower risk of incident total stroke and ischemic stroke. The lowest risks were observed in quintiles 3 and 4.

**Table 2:** Associations of incident coronary heart disease and stroke by quintiles of energy-adjusted ALA intake in 20,069 Dutch men and women^1^.

	Quintiles of ALA intake, g/d				
	Q1	Q2	Q3	Q4	Q5
N	4,013	4,014	4,014	4,014	4,014
Median ALA, g/d	1.0	1.2	1.3	1.5	1.9
Coronary heart disease					
No. events	68	51	47	53	61
Model 1^2^	1.0 (ref)	0.90 (0.63–1.30)	0.87 (0.60–1.27)	1.01 (0.70–1.44)	1.16 (0.82–1.64)
Model 2^3^	1.0 (ref)	0.89 (0.61–1.29)	0.89 (0.61–1.30)	0.97 (0.67–1.40)	1.03 (0.72–1.46)
Model 3^4^	1.0 (ref)	0.89 (0.61–1.30)	0.90 (0.61–1.33)	0.97 (0.66–1.44)	1.01 (0.66–1.54)
Total stroke					
No. events	64	43	34	35	45
Model 1^2^	1.0 (ref)	0.71 (0.48–1.04)	0.57 (0.38–0.87)	0.62 (0.41–0.93)	0.83 (0.57–1.22)
Model 2^3^	1.0 (ref)	0.68 (0.46–1.01)	0.53 (0.34–0.81)	0.59 (0.39–0.90)	0.78 (0.53–1.15)
Model 3^4^	1.0 (ref)	0.65 (0.43–0.97)	0.49 (0.31–0.76)	0.53 (0.34–0.83)	0.65 (0.41–1.04)
Ischemic stroke					
No. events	45	27	21	23	28
Model 1^2^	1.0 (ref)	0.65 (0.40–1.05)	0.52 (0.31–0.87)	0.59 (0.36–0.98)	0.74 (0.46–1.20)
Model 2^3^	1.0 (ref)	0.63 (0.39–1.02)	0.45 (0.26–0.77)	0.55 (0.33–0.92)	0.70 (0.43–1.12)
Model 3^4^	1.0 (ref)	0.63 (0.38–1.04)	0.45 (0.26–0.79)	0.56 (0.32–0.97)	0.70 (0.39–1.26)

Footnotes Table 2.

ALA: alpha-linolenic acid; Q1–Q5: quintiles.

1 Values are hazard ratios (95% CI), with the first quintile as the reference category.

2 Model 1: adjusted for age and gender (n = 20,069).

3 Model 2: model 1 with additional adjustments for body mass index, total energy intake, cigarette smoking, educational level, parental history of myocardial infarction, alcohol intake (n = 19,896).

4 Model 3: model 2 with additional adjustments for intake of vitamin C, beta-carotene, fiber, saturated fatty acids, trans fatty acids, polyunsaturated fatty acids other than ALA (n = 19,896).

Median energy-adjusted intakes of ALA from salad dressings increased from 0.1 g/d to 0.7 g/d across quintiles. ALA from other sources (mainly margarines) increased from 0.7 to 1.4 g/d. Incident CHD was not associated with ALA from salad dressings (**Table 3**) or with ALA from other sources (**Table 4**). The inverse association of ALA intake with total and ischemic stroke was stronger for ALA from salad dressings compared with total ALA, while ALA from other sources was not associated with stroke. Compared with the bottom quintile of ALA intake, participants in the higher quintiles had an 18–61% lower risk of total stroke and a 25–58% lower risk of ischemic stroke. The inverse associations were most pronounced in quintiles 3 and 4. After additional adjustment for raw vegetable consumption, the associations were somewhat weaker, but remained statistically significant, except for the top quintile of ALA.

**Table 3:** Associations of incident coronary heart disease and stroke by quintiles of energy-adjusted ALA intake from salad dressings in 20,069 Dutch men and women^1^.

	Quintiles of ALA intake				
	Q1	Q2	Q3	Q4	Q5
N	4,013	4,014	4,014	4,014	4,014
Median ALA in salad dressings,^2^ g/d	0.1	0.2	0.3	0.5	0.7
Median ALA in other sources, g/d	1.0	1.0	1.0	1.0	0.9
Coronary heart disease					
No. events	78	56	55	42	49
Model 1^3^	1.0 (ref)	0.93 (0.66–1.32)	1.07 (0.75–1.51)	0.90 (0.62–1.32)	1.20 (0.83–1.73)
Model 2^4^	1.0 (ref)	0.93 (0.66–1.32)	1.02 (0.71–1.45)	0.83 (0.56–1.23)	1.06 (0.73–1.54)
Model 3^5^	1.0 (ref)	0.95 (0.67–1.34)	1.04 (0.72–1.49)	0.86 (0.58–1.29)	1.14 (0.76–1.70)
Model 4^6^	1.0 (ref)	0.94 (0.66–1.34)	1.03 (0.71–1.49)	0.85 (0.56–1.30)	1.12 (0.72–1.75)
Total stroke					
No. events	78	60	28	26	29
Model 1^3^	1.0 (ref)	0.85 (0.60–1.19)	0.45 (0.29–0.69)	0.44 (0.28–0.70)	0.55 (0.36–0.86)
Model 2^4^	1.0 (ref)	0.83 (0.59–1.18)	0.44 (0.28–0.68)	0.41 (0.26–0.66)	0.52 (0.33–0.81)
Model 3^5^	1.0 (ref)	0.82 (0.57–1.16)	0.42 (0.27–0.66)	0.39 (0.24–0.62)	0.46 (0.28–0.74)
Model 4^6^	1.0 (ref)	0.85 (0.59–1.20)	0.45 (0.29–0.72)	0.44 (0.27–0.72)	0.57 (0.34–0.96)
Ischemic stroke					
No. events	54	37	20	17	16
Model 1^3^	1.0 (ref)	0.77 (0.50–1.17)	0.47 (0.28–0.79)	0.43 (0.24–0.74)	0.44 (0.25–0.78)
Model 2^4^	1.0 (ref)	0.74 (0.48–1.15)	0.45 (0.27–0.77)	0.40 (0.23–0.71)	0.41 (0.23–0.73)
Model 3^5^	1.0 (ref)	0.75 (0.48–1.16)	0.46 (0.27–0.79)	0.42 (0.23–0.74)	0.42 (0.23–0.79)
Model 4^6^	1.0 (ref)	0.78 (0.50–1.21)	0.50 (0.29–0.86)	0.47 (0.26–0.86)	0.51 (0.26–1.02)

Footnotes Table 3.

ALA: alpha-linolenic acid; Q1–Q5: quintiles.

1 Values are hazard ratios (95% CI), with the first quintile as the reference category.

2 Analyses on ALA in salad dressings are adjusted for ALA in other sources in all models.

3 Model 1: adjusted for age and gender (n = 20,069).

4 Model 2: model 1 with additional adjustments for body mass index, total energy intake, cigarette smoking, educational level, parental history of myocardial infarction, alcohol intake (n = 19,896).

5 Model 3: model 2 with additional adjustments for intake of vitamin C, beta-carotene, fiber, saturated fatty acids, trans fatty acids, polyunsaturated fatty acids other than ALA (n = 19,896).

6 Model 4: model 3 with additional adjustment for raw vegetables (n = 19,896).

**Table 4:** Associations of incident CHD and stroke by quintiles of energy-adjusted ALA intake from other sources than salad dressings in 20,069 Dutch men and women^1^.

	Quintiles of ALA intake				
	Q1	Q2	Q3	Q4	Q5
N	4,013	4,014	4,014	4,014	4,014
Median ALA in other sources,^2^ g/d	0.7	0.9	1.0	1.1	1.4
Median ALA in salad dressings,^2^ g/d	0.3	0.3	0.3	0.3	0.3
Coronary heart disease					
No. events	66	42	46	54	72
Model 1^3^	1.0 (ref)	0.72 (0.49–1.06)	0.81 (0.55–1.19)	0.87 (0.61–1.25)	0.96 (0.68–1.34)
Model 2^4^	1.0 (ref)	0.73 (0.49–1.10)	0.81 (0.54–1.22)	0.88 (0.61–1.29)	0.91 (0.64–1.29)
Model 3^5^	1.0 (ref)	0.73 (0.48–1.10)	0.80 (0.53–1.22)	0.85 (0.57–1.28)	0.85 (0.56–1.27)
Model 4^6^	1.0 (ref)	0.73 (0.48–1.10)	0.80 (0.53–1.22)	0.85 (0.57–1.28)	0.84 (0.56–1.27)
Total stroke					
No. events	41	38	38	45	59
Model 1^2^	1.0 (ref)	0.87 (0.56–1.36)	0.85 (0.54–1.33)	0.96 (0.62–1.47)	1.12 (0.75–1.67)
Model 2^3^	1.0 (ref)	0.91 (0.58–1.45)	0.88 (0.55–1.40)	0.97 (0.62–1.52)	1.10 (0.72–1.66)
Model 3^4^	1.0 (ref)	0.88 (0.55–1.41)	0.83 (0.51–1.35)	0.92 (0.57–1.48)	0.96 (0.59–1.56)
Model 4^5^	1.0 (ref)	0.88 (0.55–1.41)	0.82 (0.51–1.34)	0.89 (0.56–1.44)	0.93 (0.57–1.51)
Ischemic stroke					
No. events	29	26	22	26	41
Model 1^3^	1.0 (ref)	0.86 (0.51–1.47)	0.72 (0.41–1.26)	0.80 (0.47–1.36)	1.09 (0.68–1.77)
Model 2^4^	1.0 (ref)	0.85 (0.49–1.47)	0.69 (0.38–1.23)	0.77 (0.44–1.33)	1.02 (0.62–1.68)
Model 3^5^	1.0 (ref)	0.85 (0.49–1.50)	0.69 (0.38–1.27)	0.77 (0.42–1.39)	1.01 (0.56–1.83)
Model 4^6^	1.0 (ref)	0.85 (0.48–1.49)	0.68 (0.37–1.25)	0.75 (0.41–1.36)	0.98 (0.54–1.78)

Footnotes Table 4.

ALA: alpha-linolenic acid; Q1–Q5: quintiles.

1 Values are hazard ratios (95% CI), with the first quintile as the reference category.

2 Analyses on ALA from other sources than salad dressings are adjusted for ALA in salad dressings in all models.

3 Model 1: adjusted for age and gender (n = 20,069).

4 Model 2: model 1 with additional adjustments for body mass index, total energy intake, cigarette smoking, educational level, parental history of myocardial infarction, alcohol intake (n = 19,896).

5 Model 3: model 2 with additional adjustments for intake of vitamin C, beta-carotene, fiber, saturated fatty acids, trans fatty acids, polyunsaturated fatty acids other than ALA (n = 19,896).

6 Model 4: model 3 with additional adjustment for raw vegetables (n = 19,896).

The associations of ALA intake with incident CHD and stroke did not differ in subgroups of age and gender. For the subgroup with information on physical activity (n = 15,423), the full model with and without physical activity yielded similar results. Adjustment for plasma cholesterol or systolic blood pressure did not change the results. HRs (95%CI) for total stroke after additional inclusion of systolic blood pressure were 0.66 (0.44–0.98), 0.50 (0.32–0.78), 0.54 (0.34–0.84) and 0.67 (0.42–1.06) for Q2–Q5 compared with Q1, respectively.

## Discussion

In this large prospective cohort study in the Netherlands, we found no association between ALA intake and incident CHD. However, ALA intakes >1.1 g/d were associated with a 35–50% lower risk of incident stroke, mainly ischemic stroke, compared with ALA intakes <1.1 g/d.

This study has several strengths, including almost complete mortality follow up and detailed information on potential confounders. Nonfatal cardiovascular events were assessed through the national hospital discharge register. In part of the subjects included in our analysis, hospital discharge diagnoses for MI were validated by comparison with the clinical registry of the Cardiology Department of the Maastricht University Hospital, showing a relatively high sensitivity (84%) and positive predictive value (97%) for MI [35]. We assume that misclassification of stroke was limited, because brain imaging is used to identify stroke and its subtypes in 98% of the patients admitted to Dutch hospitals [36].

However, there were also limitations. First, misclassification of participants for ALA intake may have occurred. However, because we excluded participants with a history of MI or stroke, and participants who used cholesterol lowering or blood pressure lowering medication, we expect misclassification at baseline to be random rather than dependent on disease outcome. Second, hospital discharge diagnoses were assessed through probabilistic linkage with the national hospital discharge register. If we have missed events by this procedure, then this is unlikely to be related to ALA intake and may have caused bias towards the null.

In our study, ALA intakes in the range of 1.0–1.9 g/d were not associated with incident CHD. This is in line with a cohort study in elderly Dutch men (Zutphen Study), which did not show a benefit of ALA intake on incident CHD, for similar levels of intake [23]. In the Nurses' Health Study, with a difference of 0.7 g/d between the top and bottom quintile of ALA intake, ALA intake was inversely associated with fatal CHD, but not with nonfatal MI [12], [18]. Our results on CHD differ from those of the Health Professionals Follow-up Study, in which an increase of one energy percent of linolenic acid (mainly ALA) intake was associated with a 60% lower risk of incident MI in men [21]. These results were not adjusted for other PUFA, saturated fatty acids, or trans fatty acids, and the contrast between the top and bottom quintile of ALA intake was only 0.7 g/d (∼0.3 energy percent). A later study of this cohort with additional adjustment for other fatty acids suggested a 16% lower risk for total CHD (borderline significant) corresponding with an increase of ALA of 1g/d [20]. Our results also differ from a large case-control study in Costa Rica, which supported an inverse association of ALA intake with nonfatal MI, with an odds ratio (95% CI) of 0.61 (0.42–0.80) for the top (2.4g/d) vs. the bottom (1.1g/d) decile of ALA intake [13]. Similar, but stronger, associations were observed for ALA status in adipose tissue in the same study. No further risk reductions were obtained beyond the 7th decile of ALA status, corresponding to an ALA intake of 1.8 g/d [13]. Although this retrospective study suggested that benefits of ALA on CHD could already be achieved at modest levels [13], [37], our prospective study did not support this. Misclassification of ALA intake, especially within a relatively narrow range of intake, may have attenuated our associations. ALA intake levels in the Netherlands are comparable to most West European countries and the United States of America [38]. Within the range of ALA intake that we studied, the associations with CHD and stroke can therefore be extrapolated to other western populations.

Despite the small range of intake in our study, we did find an inverse association of ALA intake with incident stroke, which was most pronounced for ALA from salad dressings. It is not likely that ALA from different food sources would act differently. Although we adjusted for many potential confounders, including the intake of raw vegetables, the associations may still be influenced by a healthier diet and lifestyle of those who regularly eat raw vegetables with salad dressings. In general, correlated fatty acids in foods and residual confounding play an important role in cohort studies and results should therefore be judged with caution. Epidemiological studies of ALA intake or status and stroke are scanty. Our results are in line with a nested case-control study in middle-aged American men at high risk for CVD [25]. In that study, one SD increase of ALA in serum cholesteryl esters was associated with a 37% decrease in risk of stroke.

Humans can convert ALA into the longer-chain fatty acid EPA and eventually DHA, although conversion occurs to a limited extent [39]. Apart from potential indirect effects of ALA on CVD via conversion into EPA and DHA, ALA has been suggested to have anti-inflammatory [6], [11], anti-arrhythmic [12], anti-thrombotic [12], [13], or neuroprotective effects [14]. However, others concluded that there was insufficient evidence that ALA influences risk factors for CVD [15], [16]. Although CHD and ischemic stroke are both atherosclerotic disorders that have risk factors in common, we found differential associations of ALA intake for CHD and stroke. A proposed mechanism from animals studies for a protective effect of ALA on incident ischemic stroke is that ALA would be neuroprotective after induced ischemia, by beneficially affecting the brain blood flow [14].

Concluding, in this generally healthy Dutch population, ALA intake was not associated with incident CHD. However, the data suggested that a low intake of ALA may be a risk factor for incident stroke, although more prospective studies are needed before definite conclusions can be drawn.
